# Systematic feature evaluation for gene name recognition

**DOI:** 10.1186/1471-2105-6-S1-S9

**Published:** 2005-05-24

**Authors:** Jörg Hakenberg, Steffen Bickel, Conrad Plake, Ulf Brefeld, Hagen Zahn, Lukas Faulstich, Ulf Leser, Tobias Scheffer

**Affiliations:** 1 Humboldt-Universität zu Berlin, Computer Science Department, 10099 Berlin, Germany

## Abstract

In task 1A of the BioCreAtIvE evaluation, systems had to be devised that recognize words and phrases forming gene or protein names in natural language sentences. We approach this problem by building a word classification system based on a sliding window approach with a Support Vector Machine, combined with a pattern-based post-processing for the recognition of phrases. The performance of such a system crucially depends on the type of features chosen for consideration by the classification method, such as pre- or postfixes, character n-grams, patterns of capitalization, or classification of preceding or following words. We present a systematic approach to evaluate the performance of different feature sets based on recursive feature elimination, RFE. Based on a systematic reduction of the number of features used by the system, we can quantify the impact of different feature sets on the results of the word classification problem. This helps us to identify descriptive features, to learn about the structure of the problem, and to design systems that are faster and easier to understand. We observe that the SVM is robust to redundant features. RFE improves the performance by 0.7%, compared to using the complete set of attributes. Moreover, a performance that is only 2.3% below this maximum can be obtained using fewer than 5% of the features.

## Background and overview

Task 1A of the BioCreAtIvE evaluation [1] is a *named entity recognition *(NER) problem where entities are *gene *and *protein names *[2]. In this task, participants were given a set of 10.000 sentences with protein and gene names tagged in the text. Using these sentences, algorithms had to be designed that recognize gene and protein names in arbitrary text (in the following, we use the term "gene" as an abbreviation for "gene and protein"). The performance of the algorithms was tested based on a set of 5.000 previously unseen and untagged sentences. The evaluation was strict, *i.e.*, gene names that consist of multiple names (phrases), such as "Drosophila shc gene product" had to be recognized entirely without missing or additional parts. All sentences provided by the organizers were previously tokenized, *i.e.*, broken into tokens separated by white-spaces or sentence boundaries. Furthermore, part-of-speech information was provided in-line with the text for the training corpus.

The problems of the NER task for gene names in biomedical text have been described in detail elsewhere (for reviews, see *e.g. *[3,4]). Due to missing standards for nomenclature, genes have multiple names, abbreviations are frequent, names consist of letters, digits, and special characters, and exact phrase boundaries are often debatable. Specifically the last problem, *i.e.*, the recognition of multi-word phrases, has turned out to be a hard problem if strict evaluation is applied.

**Figure 1:**
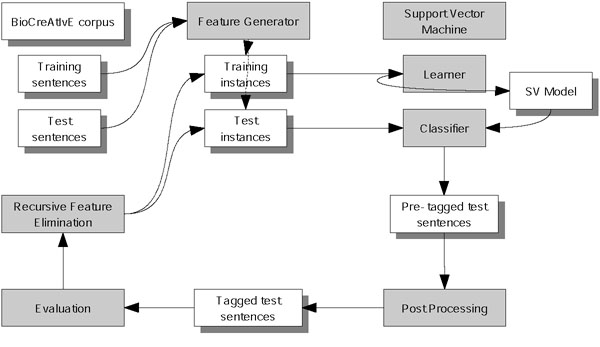
**System architecture. **The overall system architecture, including the recursive feature elimination process.

Our system essentially uses two steps for solving the NER problem (see Figure 1 for an overview of the general system architecture). In the first step, the system tries to detect at least one word of each gene phrase by using a machine learning classifier on each token. In this step, we reduce the multi-phrase problem to the decision of whether each single token is part of a gene name or not, rather than classifying complete phrases. For the machine learning classifier we represent tokens using a large set of characteristic properties (*features*), such as the whole word in itself, certain predefined character sequences (such as the suffix "-ase"), fixed-length character sequences (character n-grams, suffixes), and predefined character patterns (occurrence of special symbols, capital letters, numbers). Once a set of features is fixed, the token is represented as a feature vector indicating for each feature whether it is present in the token or not. A machine learning algorithm (in our case a *Support Vector Machine*, SVM) has to identify the set of features and their combinations that are descriptive for either gene names or non-gene names, thus finding a separation of the two classes.

In the second step, we take the classification of each single token as input and expand candidates to complete gene phrases. We call this step *post-processing*. Post-processing is based on a set of hand-crafted rules matching tokens and part-of-speech information determined by a POS-tagger. As a rule of thumb, if one word in a noun-phrase is classified as a gene name, the post-processing step tags the whole phrase afterwards.

On the BioCreAtIvE devtest corpus, our system is able to obtain a precision of 71.4% and a recall of 72.8%, corresponding to an f-measure of 72.1%, for the closed division. The same parameter setting yields an f-measure of 71.2% on the evaluation set (71.9% precision at 70.6% recall). Results for the open division are about 1% higher. Although it greatly improves the overall performance (see Results), post-processing is currently not the focus of our research. The most important reason for this is that a careful error analysis reveals that about 76% of all errors result from wrong token classifications and only 24% stem from the post-processing (see Discussion). For that reason, we focus on finding and characterizing good sets of features.

The performance of any NER system based on machine learning techniques, including classification based systems like ours and systems using sequential information (e.g. *hidden Markov models *(HMM) and *conditional random fields *(CRF) [5-8]), crucially depend on the "right" set of features. Having too large a feature set may degrade system performance both in terms of quality of predictions as in terms of execution time. The feature set we used for the BioCreAtIvE evaluation was determined using a laborious and manual trial-and-error approach. The same, essentially heuristic and intuition-driven approach was apparently used by other systems in the contest as well [9,10], but it cannot guarantee the best possible set of features. Our focus in this paper lies on the systematic evaluation and ranking of different feature classes and their combinations.

Clearly, it is not feasible to test all subsets of more than 250.000 features, which is the total number of features our system can generate. We therefore follow a *recursive feature elimination *(RFE) [11] procedure that successively removes the features with the smallest impact from the training samples, followed by a re-evaluation of the resulting new model (see Figure 1). Using RFE, we achieve a number of different goals. First, RFE provides a ranking of features regarding their importance for learning the best model possible. We thus hope to improve system performance by removing non-descriptive features. Second, it helps to understand and gain insights into the model, separating helpful from malicious features. This generates hints into which direction future development should look. Third, the resulting system is much smaller in terms of the number of features used, leading to systems with better time performance.

Note that even using RFE as we did does not guarantee to find the truly optimal set of features, as many combinations remain untested. In this sense, RFE is still a heuristic for determining a hopefully best candidate set.

In the following section we present results of the feature engineering and elimination. After that, we discuss our results, provide a detailed analysis of the errors of our system, and compare it to other systems. The Methods section describes our system and the engineering process in detail.

## Results

The system we implemented for the BioCreAtIvE evaluation used a set of feature classes determined by trial and error. We shall first present results for this system. After the competition, we applied a more systematic method for finding the optimal set of features. For this approach, we started with a model using the full set of all feature classes and recursively removed the features having the lowest weights in the model learned by the SVM. The results are presented in the second section of this chapter. Finally, we show the impact of our post-processing step, which was the same in both cases. The set of all features considered in either approach, together with their impact on a baseline classifier, can be found in Table 1.

### Original feature sets

For the BioCreAtIvE submission, we implemented a number of different feature classes and checked a variety of combinations in which we toggled the usage of classes on and off. The subset of feature classes that we used in the final submission is marked "*" in Table 1. As a baseline, the system yields an f-measure of 54.1% when only the tokens themselves are used as features. As an example, after adding character 1-, 2-, and 3-grams, the system reaches 68.2%.

We observed that using all features leads to lower performance. We thus defined a particular subset of surface clues yielding the best f-measure we could measure. This subset includes the tests for a single or two capital letters that comprise a token, only capital letters in a token, a mix of lower and upper case letters only, occurrence of special characters and combinations of letters and digits. Possibly due to the correlation of features classes, not all of these features improved performance when chosen alone, but only in combination with others. For instance, special characters and letter/digit combinations improved performance only when used together.

The competition had two divisions, one called *open*, allowing for the construction of gazetteers from arbitrary data sources, and one called *closed*, where gazetteers could only be built from the training data provided by the organizers. This separation was introduced since one expects a larger impact of gazetteers. Interestingly, this has not turned out to be true. Gazetteers built automatically showed a rather low impact when used in addition to the gazetteers derived from the training data. When we took as features only the tokens and the gazetteer built from training data and external sources (synonym lists for gene names from mouse, yeast, and fruit fly (all from BioCreAtIvE Task 1B), and human [12]), performance drops slightly, compared to taking only tokens as features (see Table 1). When gazetteers were used together with other feature classes we observed a minor improvement; therefore, the respective gazetteer was used in our submissions for both divisions. As the overall results showed, there was only a 1% difference between open and closed division. The small gain in performance is in contrast to the results from Task 1B, where the best performing systems were largely based on manually curated dictionaries.

Another interesting feature class are part-of-speech tags. As Table 1 shows, using only tokens and the part-of-speech tags provided with the data lets the f-measure drop to almost zero. The feature vector in this case consists of one feature for each token in the training corpus and features for each part-of-speech tag (we used 12 different tags). In this vector, only two entries are 1 (the feature representing the token itself and the part-of-speech tag of the token). Apparently, this information completely confuses the SVM. However, other systems showed that using POS when trained on a domain-specific corpus and used in combination with other features can improve the performance of NER systems [5,6].

### Feature engineering / recursive feature elimination

In the aftermath of the BioCreAtIvE workshop, we improved our system in two ways. First, we added a number of new features, partly inspired by other participants of the challenge, partly taken from literature or our own inspiration. This includes character 4-grams, a feature checking for tokens starting with lower case letters followed by a mixture of lower and upper case, and special features for Greek letters and Roman numbers (see Table 1; new features are marked "◦"). A check for Greek letters increases the performance slightly, Roman numerals add to the precision of our system. Combined, all new features add about 2% to the f-measure when added to the previously best subset of features. The most successful subset of all features (new and old) that we have been able to identify manually so far performs about 4% better than the full feature set, and about 2% better than our submission to the BioCreAtIvE evaluation. Second, we tried to find a more systematic way of finding and characterizing sets of features. The heuristic selection of feature classes performed for the BioCreAtIvE evaluation is unsatisfactory for a number of reasons. First, one cannot know whether other combinations of features would yield better results. Second, we chose the set of features by dropping only entire feature classes, but not single features, since this seemed infeasible. Third, the time necessary for learning the model and for using the model for prediction is very high for large sets of features.

Therefore, we studied the impact of a gradual exclusion of features. We start with a full model containing all features described in Table 1. We iteratively remove a number of the features with the lowest weight, retrain the model, and check the resulting performance; this procedure is referred to as *recursive feature elimination *(RFE). Post-processing was turned off during these runs, as the decision about removal of features is based solely on the absolute value of the weight vector resulting from the SVM training. We expected the RFE to (a) remove features and feature classes decreasing the performance of our system, and to (b) lead to a concise and informative model in the end.

**Figure 2:**
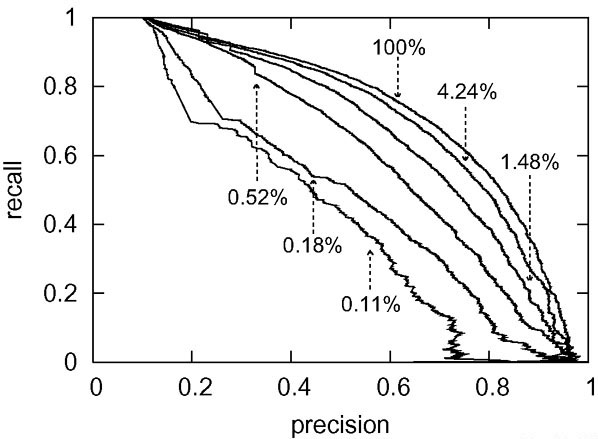
**Impact of the Recursive Feature Elimination. **Impact of removing 10% of the features with the lowest weight vector in each round. After 30 iterations, with only 4.28% of all features remaining, the f-measure has dropped only by 2%. The underlying evaluation method only considers the recognition of single tokens rather than whole phrases. The bottom line (65 iterations) shows the impact of the remaining 0.11% of all features. All values are evaluated without the post-expansion step (see text).

**Figure 3:**
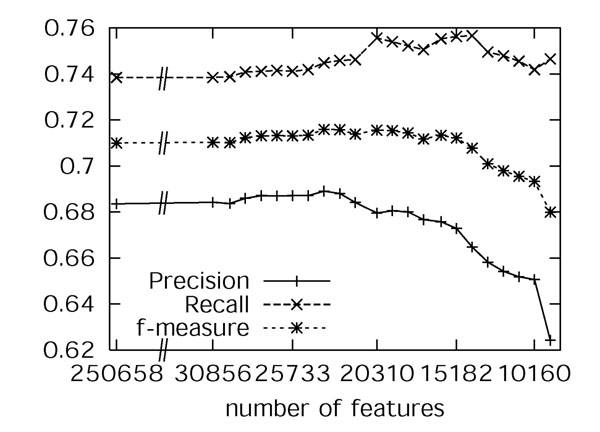
**Dependence of the f-measure on the number of features. **Performances (precision, recall, f-measure) for different numbers of features. Starting from the full feature set, recursive feature elimination removes the features with the lowest weight vector and we measure the performance after each round.

Regarding (a), we made two experiments. First we removed the 10% features with lowest weights in each iteration. Figure 2 shows the correlation between removal rates and performance. Figure 3 shows the change in performance corresponding to the remaining number of features. We see that the performance drops slowly, but constantly with each iteration. Interestingly, we find that even after removing more than 95% of the features, the performance drops only marginally (by about 2.3%). In a second experiment, we reduced the step-width in each iteration using the following scheme. We removed the 1.000 features with lowest weights and all features with zero-weight in each round. RFE achieves a maximal f-measure of 71.1%, when approximately 23.000 features remain (see Figure 3).

Regarding (b), we performed an RFE until only 150 features remained. These 150 (presumably most discriminating) features can be divided into the following classes: about 70 token features, about 70 different 3- and 4-grams, the gazetteer feature (whether or not the word appears in the dictionary), and nine different surface clue features (allCaps, initCaps, combi, special_upperCase, special_number, greek, lowerUpper, singleCap, capMixLetters – see Table 1 for examples). Table 2 shows a list of examples from all classes including their weights.

The 70 single token features again can be roughly split into three groups: common stop words receiving a highly negative weight (such as "the" or "are"), common important keywords receiving a highly positive weight ("kinase", "protein"), and abbreviations ("Gnt", "ZII") receiving either positive or negative weights. Examples for the 70 remaining 3- and 4-grams are "ing" and "ese" (popular suffixes), or "76-k", "Stai", and "GTTA". Interestingly, only very few character 3- and 4-grams overlap each other, indicating that the RFE has effectively resolved the obvious correlation in those cases. To find patterns in the most important n-grams, we stopped a new RFE run as soon as only 500 n-grams remained. We then counted their occurrences in the whole corpus, finding that most of them occur more often in gene names than outside or vice versa. For example, the 4-gram "nRNP" occurs 20 times in the corpus (denoting an abbreviation for "nuclear ribonucleoprotein") in words like "hnRNP" or "snRNP-specific", all of which are (parts of) gene names. Behind most highly ranked token features we can find biological meanings, as in the previous example. On the other hand, the 4-gram "oped" (e.g., part of "developed") has a negative weight and occurs exclusively outside gene names (55 times).

### Post-expansion to complete gene names

The expansion of the single tokens predicted by the SVM to full gene name phrases uses a set of hand-crafted rules (see Table 3). Details on the structure of our rules can be found in the Methods section. These rules either extend a, possibly unconnected, sequence of tokens to a full phrase, or remove tags from isolated tokens. In sequences of tokens tagged as gene name by the SVM with a length larger than one, positive tags are never removed.

**Figure 4:**
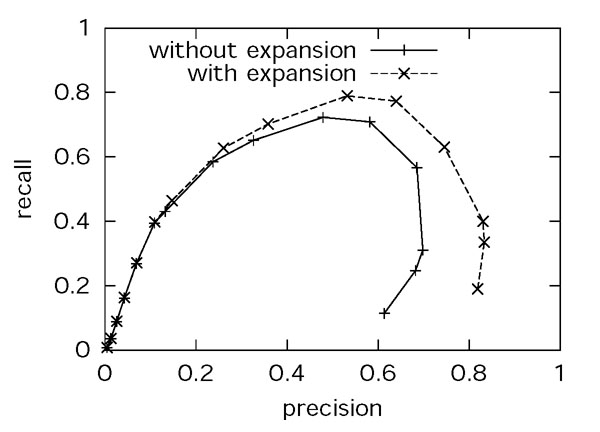
**Recall/precision with and without post-expansion. **Comparison of recall and precision before and after the post-expansion step. We use the full feature set (marked "100%" in Figure 2) for this evaluation. We obtain the different spots by parallelly shifting the hyperplane.

Post-processing has a high impact on the performance of our system, adding 12% precision and 10% recall compared to the original system. It corrects about 300 previously incomplete gene names (more than 20% of the former false negative predictions). It additionally removes 120 false positives (10%) by explicitly deleting generic single word names (*e.g.*, "protein"). By manual inspection we found the post-processing never makes a mistake in the latter cases. In Figure 4, we present the impact of the post-expansion on recall and precision.

## Discussion

Our system for the recognition of gene and protein names in natural language text conceptually has three blocks: First, a number of features are defined that can be advantageous for the classification of each word in isolation. Second, we use a recursive feature elimination to find the best set of features, *i.e*., those features that offer the best performance for the machine learning classifier doing the actual classification. Third, we use a rule-based post-processing to combine classifications of single words into classification of phrases. We will discuss each of these topics, starting with the impact of the RFE step. The general set of features and their impact will be discussed based on a comparison to other systems in the related work section.

### Recursive feature elimination

Application of RFE has been beneficial in several ways. It helped to track down the set of most important features. Most notably are the remaining keyword tokens. These are tokens appearing very often inside gene names or at least collocated with them. As the tokens form common words, they lack descriptive n-grams or other properties such as upper case letters, and thus have to be represented by themselves. According to intuition, the common keywords described above have a positive weight, common stop words a negative weight. Another positive aspect of RFE when applied in a fine-grained manner is that it helped to improve the system's overall performance (see Figure 3). Although our currently best system was still found by manual combination of feature classes, RFE is already quite close to this performance. Thus, applying RFE from the beginning of a project can help to drastically reduce the amount of time invested into trying different combinations of feature classes. A final positive aspect is that we discovered that only 5% of all features perform almost as well as the whole set in discriminating gene names from other words (see Figure 2). This can save considerable time when applying NER to a large corpus such as the entire MEDLINE.

### Related work

Some groups participating in the BioCreAtIvE evaluation 2003 make use of techniques similar to ours. Three other groups propose token-based classifiers with SVMs. The performance of such approaches varies widely. In the following, we will present the systems most similar and compare the approaches to ours.

### PosBIOTM-Ner

Song *et al*. [9] use a modification of the classes separating gene names from other words to the BIO-markup. This includes not only the differentiation between tokens inside and outside a gene name (I/O), but they add a class for tokens at the beginning of a compound gene name (B). Our own system only uses the I and O classes. A significant contribution to the feature space is a gazetteer lookup for tokens and phrases based on edit-distances. The edit-distance does consider lexical variations. In our own approach, we use exact matches of candidate terms, but against a gazetteer containing spelling variants. Song *et al*. try to enlarge the training sample artificially. The system reuses sentences from the training corpus with replaced gene names. Assuming that every named entity appears only in base noun phrases, all tokens outside noun-phrases plus determiners are excluded completely. The method scores an f-measure of 73.8% without and 66.7% with the additional training examples, indicating that the restriction to noun-phrases does not improve the overall performance. The recall is about 1–2% higher when using artificial examples, and precision drops by 17%. BIO-classes and the invocation of a gazetteer are the main differences from our system, and PosBIOTM-NER scores about 1% higher f-measure, mainly because the predictions are more precise (80% precision at 68.5% recall).

### YamCha

Mitsumori *et al*. [10] use the same extension to BIO-classes as the system presented above. The method additionally uses a context window, which, however, does not improve the performance of our own system. We also experimented with context windows (symmetrical and asymmetrical) of different sizes, but did not find positive influence on the overall performance. However, note that context is implicitly used by our post-processing step. SWISS-PROT and TrEMBL data comprise the gazetteer feature used in YamCha. It enhances performance by 3% up to an f-measure of 78.1%. In addition to the different source for gazetteer entries, YamCha makes use of the gazetteer in a different way than we do. Token n-grams are used for exact matches against phrases in the gazetteer. YamCha lacks a name expansion step but performs about 5% better than our system. This is surprising, as the performance of our system without post-processing is about 15% less than with post-processing.

### PowerBioNE

Zhou *et al*. propose an ensemble methods of two HMM and one SVM classifier [5,6]. The SVM uses a feature set similar to ours. Rather than having one feature representing the appearance of a candidate token in a vocabulary, PowerBioNE has one dimension for each word in the vocabulary. Orthographic features for the representation of tokens are comparable to ours, but include checks for parentheses, punctuation, and stop words as well. Our own implementation consists of the 100, 1,000, and 10,000 most common English words, and we do not know about the composition of PowerBioNE's stop word list. Zhou *et al*. distinguish two kinds of triggers indicating whether a token is included in two separate lists of words. One consists of words typically occurring inside gene names, and the other contains words typically found in the local context of gene names. Our own system measures the distance to such keywords and sets the value for one single feature according to this distance. However, as the authors propose an ensemble method, the exact difference in prediction performance compared to our system is not known. The same is true for the influence of different feature sets.

Some of the features described by other participants as advantageous do not enhance (and sometimes even decrease) our prediction performance. We reproduced all features which were not in our original system, but most of them do not contribute to an improvement of our system (e.g., *ldl*, *ddd*, *DNA *– see Table 1). The recursive feature elimination removes almost all of them in early rounds, indicating that their discriminative power is low.

Surprisingly, most features we use to train the Support Vector Machine, and which invoke context information, did not demonstrate their presumed value. This result is in contradiction to other systems, which benefit from similar features. Even information on the (predicted) class of the neighboring tokens does not improve our results. This method increases precision by approximately 6%, but reduces recall by approximately 10%. Our post-processing phase adjusts for this. It uses the local context, based on part-of-speech information, to expand predicted tokens to compound names. The most successful systems presented for the BioCreAtIvE evaluation use sequence-based NER systems rather than pure SVMs. Those systems depend on the engineering of a carefully chosen set of features as well. We therefore believe that our results can be helpful for improving those approaches, too.

Other systems based on SVM classifiers on similar problems have been proposed before. They were evaluated on different corpora, however, and performances may not be entirely comparable. GAPSCORE [13] scores single words based on a statistical model of gene and protein names that quantifies their appearance, morphology and context. The authors compared naïve Bayes, maximum entropy, and SVM classifiers, and found that the latter outperforms the others slightly. Detected gene name candidates are extended to preceding and following tokens based on POS information. GAPSCORE achieved an f-measure of 57.6% on the YAPEX corpus for exact matches.

The system described by Seki et al. [14] uses surface clues to detect potential protein name fragments, and additionally applies a false positive filter. A probabilistic model expands single names to compounds. For exact matches, the system achieves an F-measure of 63.6% on the YAPEX corpus.

We reproduced most of those features proposed in the BioCreAtIvE evaluation, which were not included in our initial submission. We discovered that some of them (character 4-grams, a feature checking for tokens starting with lower case letters followed by a mixture of lower and upper case, and special features for Greek letters and Roman numbers) help to improve the performance of our own system. The gazetteers of all systems were implemented in different ways. It would certainly help to check for the most successful implementation, because impacts were not measured by other groups. As we learn from the other contributions, a modification of the binary classification to the B-I-O-classes seems to be a clever idea to improve prediction quality.

### Error analysis

**Figure 5:**
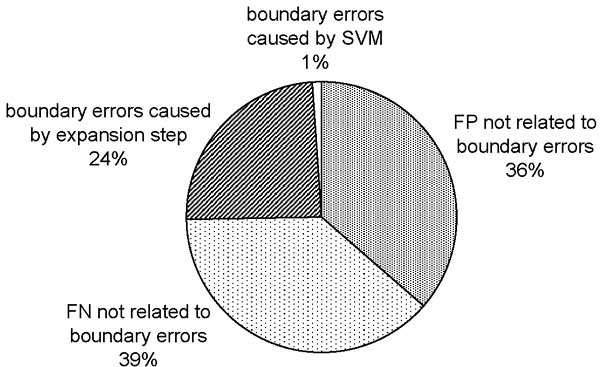
**Error analysis. **Proportions of different causes for four classes of errors. We distinguish between boundary errors and non-boundary errors (see text).

We conducted an analysis on where and how our system fails on the evaluation data set. Figure 5 shows the distribution of the errors. Boundary errors are cases where we correctly tagged at least one token of a gene phrase consisting of more than one token but we either did not tag the complete phrase or we did tag too many tokens exceeding the boundary of the phrase. 25% of all errors are boundary errors. Only 1% of those are caused by the SVM, *i.e.*, cases where the SVM falsely predicted a token as gene name that lies adjacent but outside a gene phrase. All other boundary errors are caused by the post-processing by wrongly including tokens into phrases or excluding tokens from phrases.

We also categorized the boundary errors into four types. In 27% of all boundary errors, the tagged sequence exceeds the real boundary to the left, and in 28% the real phrase starts left of the predicted boundary. In 32% of the cases, the tagged sequence exceeds the real boundary to the right, and in 31% the real phrase ends after the predicted boundary on the right. Note that those percentages do not sum to 100% because the errors at the left and right boundary can be present at the same time in one gene name. Overall, the errors of the expansion step are almost balanced regarding the direction (left/right) and also the type (exceeding/insufficient tagging).

The other errors not related to boundary errors divide into false positives and false negatives (Figure 5). These are cases where predicted gene tokens do not even overlap with an actual gene token sequence. Such errors must can traced back to our first step, the classification of single gene tokens, since post-processing only extends token sequences or removes single tags from single token sequences, but never makes mistakes in the latter cases. Together with the 1% boundary related errors, we account 76% of the errors to the single token classification by the SVM. Only 24% are caused to the expansion step. Finally, we found that our system predicts many false positives which are very generic terms, such as "transcription factor" or "rat gene". The BioCreAtIvE task definition excludes these names from being gene names.

## Conclusion

We have presented a system for recognizing names of genes in proteins in text. Our approach is a word classification system based on a sliding window approach with a Support Vector Machine. We combine this prediction of candidate tokens with a pattern-based post-processing for the recognition of compound names. We found that the most important features and feature classes for discriminating gene names from other text are the tokens themselves, character n-grams, a gazetteer, and orthographic features. Patterns checking for upper case letters, digits, or special symbols and mixtures, and Greek words comprise the latter. Distances to keywords completed this set.

We showed that a careful selection and test of feature classes used for vector space model representation of tokens has a significant impact on performance. It is not only crucial to gather a variety of feature classes, but equally important to check them for possible negative influences. For the NER problem at hand, feature selection did have an influence on the prediction quality. We observed that recursive feature elimination detects at least some of the features and feature classes having a negative influence on the performance. RFE is able to detect a small subset of all features, which performs similarly well as the full set.

## Methods

The system we implemented is a composition of two steps. A Support Vector Machine classifier [15-17] is trained on predicting whether a single token is part of a gene name or not. Our system furthermore invokes a set of context-sensitive post-processing rules that label certain surrounding tokens as genes to detect multi-word names [18].

The SVM uses a vector space representation of examples. Each feature of an example corresponds to a dimension in the vector space, and has a parameter value characteristic for each example. In our case, every token in the training and test data forms an example. All examples are labeled either as positive or negative (part of a gene name or not). The SVM learns a discriminating margin hyperplane between the two classes, described by *Support Vectors*. The decision whether a new example (from the test corpus) gets a positive or negative label then depends on the side of the hyperplane the example lies on.

### Definition of features and feature classes

For each sample token from the training and test corpus, we generate a set of features from the token and its context. We toggle the usage of whole feature classes rather than single features, e.g. all character 2-grams and not only "al", "lp", or "ph". Feature engineering thus concentrates on identifying the most helpful feature classes (see Table 1 for examples). We generate the features according to the following definitions of different feature classes:

### Token, unseen token

We take all tokens encountered in the training set as single features. If they apply to an example, they are set to their respective *tf.idf *values. We want to learn a model that is robust to new tokens. To achieve this, we introduce a single binary feature ("unseen"), and set it to 0 if the token appears in the training set, and to 1 otherwise. During the training phase, we randomly treat tokens as unseen. This means, we set the "unseen" feature to 1 with a small probability, and do not set the proper *tf.idf *value for the single token feature.

### Character n-grams of tokens

We extract substrings of length one to five from all tokens in the training set, each representing a single, *tf.idf*-weighted feature.

### Gazetteer

We match tokens and phrases against a gazetteer. The latter is built from the training corpus and (for the open division) additional synonym lists from four organisms (human, yeast, mouse, fruit fly). The gazetteer ignores capitalization, and uses wildcards for digits, and word separators (spaces, hyphens, slashes).

### Surface clues

We add binary features to the vector space based on the composition of tokens. These features represent matches against various patterns composed of letters, digits, and special characters (see Table 1).

### Keyword distance

We measure the distance of an example to the nearest keyword. Keywords are 25 hand-crafted terms, such as "receptor", or "kinase", and were deduced from the training corpus.

### Post-processing for name expansion

We employ a post-processing step in which we apply simple hand-crafted rules to expand names, because many gene names consist of multiple words. We find that the SVM classifier recognizes these sometimes incompletely – frequently, unseen nouns and unseen or nondescript adjectives are missing in predicted names.

The rules refer to the tokens, their labels generated by the SVM, and their POS tags. For the latter, we use POS tags taken from the Brill tagger [19]. This tagger uses its default lexica and contextual rules, learned from unspecific data, namely the Wall Street Journal corpus [20], and the Brown corpus [21]. As a rule of thumb, when a noun phrase contains a gene name, then the whole noun phrase is typically a gene name – except for a number of special nouns and adjectives which are never or rarely part of a gene name. We gather these nouns and adjectives from the tagged training corpus by collocation analysis. Every word appearing more frequently as a part of a gene name than preceding a gene name (and vice versa) is included in a NEWGENE phrase. This leads to the application of two exclusion lists for nouns – we expand a phrase, if the noun is none of 372 or 222 particular terms, respectively, (see Table 3). We decided to use a negative list, as we found much more nouns included in gene names than appearing directly before of after such a name. With adjectives, it is quite the opposite, though. Adjectives may be parts of gene names or qualify a gene. We expand a phrase to include a preceding or following adjective, on the other hand, if the latter appears in a list of 778 particular terms.

The rules consider formations of gene names around "/" and parentheses, too. As an additional false positive filter, we remove 23 single words tagged as NEWGENE, that are rarely gene names when occurring by themselves. Examples for such words are "alpha", "gene", "mRNA", and "subunit".

### Recursive feature elimination

In the following we briefly describe recursive feature elimination [11]. In a linear SVM, a linear decision function classifies a new token with the feature vector *x *by the sign of , where *w *is the weight vector and *b *an additive threshold. Disregarding the latter, the inner product denotes a linear combination of the features of *x*, where each feature is weighted by its corresponding component of *w*. Since we allow only positive feature values, the sign of each component of *w *determines if the corresponding feature is an indicator for (positive sign) or against (negative sign) a gene name. Their absolute values are equivalent to their impact on the inner product and therefore on the decision. Each RFE iteration starts with training and evaluating the SVM classifier. Afterwards, we firstly remove all redundant features having a zero component in *w*. Secondly, we eliminate in different experiments either a fixed percentage or a fixed number of the remaining features, respectively. These features are chosen according to their corresponding lowest weights of *w*. The next RFE iteration then begins with retraining on the reduced feature set.

Applying this elimination after training the classifier recursively should lead to a small set of relevant features that may be beneficial for further investigations. The main advantage is an easier interpretation of the computed solution, due to the reduced dimensionality.

## List of abbreviations

CRF – conditional random fields; HMM – hidden Markov model; NER – named entity recognition; POS – part of speech; RFE – recursive feature elimination; SVM – support vector machine.

## Authors' contributions

CP implemented most of the pre-processing, feature generation and classification system. SB implemented and evaluated the gazetteer lookup. JH wrote the post-processing engine and some feature generators. UB and HZ were responsible for the recursive feature elimination process. The error analysis was done by LF, SB, and JH. UL and TS provided many of the ideas for features and methods for evaluating the system. The manuscript was written by JH and discussed by all authors. All authors have read and approved the final manuscript.

## Figures and Tables

**Table 1:** Feature classes and their impact prediction quality. Table of all feature classes. *: classes used in the BioCreAtIvE submission, ◦: classes implemented afterwards, partly adopted from other participants of the contest. The forth column gives the impact of each single feature class compared to the baseline (only tokens). This figures include post-processing. The fifth column shows which how precision and recall are affected. *Letter surface clues *(last rows) refer to the following features: {special, allCaps, initCap, capMix, lowMix, Idl, ddd}.

Feature	Example	Short name	Impact	
Token*	Sro7	Token	= 54%	- *baseline *-
Unseen token*		UToken		
n-grams of token*		1G, 2G, ..	+15% +14%	1..4-grams, P+, R++ 1..3-grams
Previous & next tokens		P/NToken	-5% -6%	[1,1]-window, P+, R- [2,2]-window
n-grams of tokens in window		2PG/2NG/..		
Prefixes, suffixes		1P, 2P, 3P, 1S..	±0	
Stop word	the, or	Stop	-5% -1% -.5%	10,000 words, P+, R- 1000 words, P+, R- 100 words, P+, R-
POS tag	NN, DT	POS	-50%	P-, R-

Initial upper case*	Msp	initCap	+.5%	P=, R+
All chars are upper case*	MMTV	allCaps	+.5%	P-, R+
Upper case letters*	InlC, GUS	Upper		
Upper case (skip first)*	MsPRP2	Upper2		
Single capital	A	singleCap	+.5%	P+, R+
Two capitals	RalGDS	twoCaps	+.5%	P+, R+
Capital, then mixed letters ◦	IgM	capMix		
Lower case, then mixed ◦	kDa	lowMix	+1%	P-, R+
Special symbols*	ICAM-1	special	±0	P-, R+
Characters and numbers*	p50	CharNum		
Numbers*	p50, HSF1	Number		
Letters, digits, letters ◦	H2kd	Idl	±0	
Digit, dot, digit ◦	5.78	ddd	-.1%	P-, R-
Greek letter ◦	alpha	greek	+.5%	P+, R-
Roman numeral ◦	II, xii	roman	±0	R+, R-
Number followed by '%' ◦	75.0%	percentage	-.1%	P-, R-
DNA, RNA sequences ◦	ACCGT	DNA, RNA	-.1%	P-, R-
Longest consonant chain *	Sro7 → 2	LCC	-2%	P-, R-

Keyword distance*		keyDist	-20%	P+, R-

Gazetteer*		Gaz	-3%	P-, R-

Prev./next token is NEWGENE		PTG, NTG	-18%	prev. only, P+, R-

Tokens + letter surface clues			+2%	P+, R-
Tokens + 1,2,3-grams + greek + roman + letter surface clues			+14%	P+, R++
Tokens + 1,2,3,-grams + keyDist + Gaz + LCC + special + combi + allCaps + initCap *			+16%	P+, R++
Tokens + 1,2,3,4-grams + keyDist + Gaz + LCC + special + combi + allCaps + initCap* + lowMix ◦			+18%	P+, R++

**Table 2:** Feature classes remaining after the RFE. Examples for features and feature classes remaining after 64 iterations. In every round, we remove the 10% of all features having the lowest weight. After the 64 iterations, only 0.12% of all features remain. We show the upper, middle, and lower weighted features in this table. High weighted features are more likely to apply to positive samples (NEWGENE), low weighted features to negative samples. Names in bold indicate binary orthographic features and the gazetteer (Gaz), in contrast to single features, like a particular 3-gram. The feature named *special *in Table 1 actually consists of four parts, two of which are present in the list of top ranking features.

Feature	Class	Weight	Feature	Class	Weight
	**Gaz**	1.497386	AACC	4-gram	0.088738
insulin	Token	0.632708	D2-m	4-gram	-0.022443
protein	Token	0.628168	Stai	4-gram	-0.082046
kinase	Token	0.608392	mig	3-gram	-0.083135
human	Token	0.536695	Reve	4-gram	-0.096548
proteins	Token	0.535368	ing	3-gram	-0.099499
	**greek**	0.498111	GnT	Token	-0.099619
	**combi**	0.489201	owl	3-gram	-0.100996
serum	Token	0.480326	231	Token	-0.104751
	**lowerUpper**	0.457806	ZII	Token	-0.105133
	**singleCap**	0.438028	had	Token	-0.106545
factor	Token	0.438028	we	Token	-0.107104
wild-type	Token	0.389359		[..]	
	**initCaps**	0.366269	that	Token	-0.174203
mutants	Token	0.340689	scre	4-gram	-0.175351
genes	Token	0.340352	OH	Token	-0.179445
promoter	Token	0.327395	ims	3-gram	-0.182513
receptor	Token	0.323412	be	Token	-0.186265
polymerase	Token	0.305972	.	Token	-0.188904
complex	Token	0.292019	To	Token	-0.189576
receptors	Token	0.292019	acyc	4-gram	-0.191766
c-myc	Token	0.292019	the	Token	-0.192838
sites	Token	0.243349	off	Token	-0.197588
mutant	Token	0.243349	rank	Token	-0.198915
domain	Token	0.231541	Dar	Token	-0.205479
sequences	Token	0.216691	(	Token	-0.206405
sequence	Token	0.216683	omit	4-gram	-0.220064
domain	Token	0.215116	nost	4-gram	-0.223077
	**specialnumber**	0.205077	spit	4-gram	-0.238335
isoforms	Token	0.194679		**allCaps**	-0.243183
	**specialupperCase**	0.179926	oped	4-gram	-0.246457
	**capMixLetters**	0.179394	The	Token	-0.246535
	[..]		aged	Token	-0.253814
lare	4-gram	0.105354	are	Token	-0.267228
bicu	4-gram	0.103185	ssif	4-gram	-0.272211
bea	3-gram	0.100539	encoding	Token	-0.447471
[	Token	0.097113	which	Token	-0.535368
ntei	4-gram	0.093310	activate	Token	-0.535368
GTTA	4-gram	0.088738	contain	Token	-0.640844

**Table 3:** Rules used for the post-expansion step. The rules switch certain part-of-speech tags to NEWGENE tags. We exclude 372/222 nouns from the expansion, and include only 778 particular adjectives in the expansion of noun phrases. NN*: nouns, proper nouns, plurals; JJ: adjective; CD: cardinal digit; DT: determiner; '/' refers to the token itself.

Former POS pattern	Expanded pattern	Limitation
NEWGENE NN*	NEWGENE NEWGENE	all but 372 particular nouns
NN* NEWGENE	NEWGENE NEWGENE	all but 222 particular nouns
JJ NEWGENE	NEWGENE NEWGENE	only 778 particular adjectives
NEWGENE JJ	NEWGENE NEWGENE	only 778 particular adjectives
NEWGENE DT NN*	NEWGENE NEWGENE NEWGENE	
NEWGENE CD	NEWGENE NEWGENE	
NN* / NEWGENE	NEWGENE NEWGENE NEWGENE	
NEWGENE / NN*	NEWGENE NEWGENE NEWGENE	
